# Measuring Driver Perception: Combining Eye-Tracking and Automated Road Scene Perception

**DOI:** 10.1177/0018720820959958

**Published:** 2020-09-29

**Authors:** Jork Stapel, Mounir El Hassnaoui, Riender Happee

**Affiliations:** 1 541087 Delft University of Technology, Netherlands

**Keywords:** situation awareness, automated driving, SAGAT, ADAS, gaze, driver support

## Abstract

**Objective:**

To investigate how well gaze behavior can indicate driver awareness of individual road users when related to the vehicle’s road scene perception.

**Background:**

An appropriate method is required to identify how driver gaze reveals awareness of other road users.

**Method:**

We developed a recognition-based method for labeling of driver situation awareness (SA) in a vehicle with road-scene perception and eye tracking. Thirteen drivers performed 91 left turns on complex urban intersections and identified images of encountered road users among distractor images.

**Results:**

Drivers fixated within 2° for 72.8% of relevant and 27.8% of irrelevant road users and were able to recognize 36.1% of the relevant and 19.4% of irrelevant road users one min after leaving the intersection. Gaze behavior could predict road user relevance but not the outcome of the recognition task. Unexpectedly, 18% of road users observed beyond 10° were recognized.

**Conclusions:**

Despite suboptimal psychometric properties leading to low recognition rates, our recognition task could identify awareness of individual road users during left turn maneuvers. Perception occurred at gaze angles well beyond 2°, which means that fixation locations are insufficient for awareness monitoring.

**Application:**

Findings can be used in driver attention and awareness modelling, and design of gaze-based driver support systems.

## Introduction

Perceptual errors contribute 76% of situation awareness (SA) errors (Jones & Endsley, 1996) and are among the most frequently reported causes for accidents at intersections, which represent 20% of European road accidents (European Road Safety Observatory, 2018). Vehicles are becoming more aware of their surroundings. Machine perception can locate road users through detection and classification systems (Kooij et al., 2014; Liu et al., 2016). It processes raw sensor data in a series of filters trained to extract features, which collectively capture the concept of an object category. However, machine perception generally does not outperform human perception. Since the filters are trained from examples, they only function reliably in conditions similar to the training set. They also cannot comprehend what is seen, and only indicate if an object class occupies a particular region in the image. On the other hand, machine perception has superior attention in detection tasks. It can process the entire road scene without constraining to a region to attend, and does not suffer from vigilance decrement or biases from expectations. Machine perception can therefore support drivers in perceiving relevant road users through auditory and visual notifications. However, our senses receive more information than we can process with undivided and optimal fidelity, which we only overcome with a keen ability to be selective in what to attend. Augmentation of this process can only complement the driver effectively when it is equally selective, and becomes available well before the need is evidenced by a driver’s inaction. To achieve this, driver support systems have to identify discrepancies between what is and what should be attended.

While considerable progress has been made in the development of systems to judge object relevance (Gao et al., 2019; Gary & James, 2019) or to redirect attention using audio (Ho & Spence, 2009), augmented reality (Kim et al., 2018), and peripheral displays (Yang et al., 2018), a key challenge lies in the decision when drivers need to be warned. Current systems rely on heuristics like “only alert when dangerous, rare or in conflict with common expectation,” which generally limits operation to immediate hazards. Targeted support for developing hazards or noncritical lapses can only be achieved when driver awareness toward individual road users is monitored.

Eye tracking seems to be an ideal method to monitor what drivers have seen or overlooked, since people tend to fixate at what they inquire information from. de Winter et al. (2018) showed that glance behavior correlated better with supervision performance than the popular Situation Awareness Global Assessment Technique (SAGAT). Meghanathan et al. (2019) demonstrated that refixation patterns can discriminate encoding and memorization activity, and indicate change detection performance. However, fixation location does not always correspond to what is processed cognitively (Endsley, 1988; Rumar, 1990). Peripheral vision can suffice for lane keeping (Summala et al., 1996) and hazard detection (Huestegge & Böckler, 2016). Conversely, we can fail to see things we fixate on (Mack & Rock, 1998), but it is yet unknown how frequently drivers miss other road users despite fixating upon them or how to infer this from gaze behavior.

While aggregate metrics like distraction or fatigue have been inferred from vehicle-fixed regions of interest or direction independent measures like gaze variance (Rendon-Velez et al., 2016), gaze-based awareness classification of individual objects remains an open challenge. Attention prediction models like top-down saliency maps (Xia et al., 2019) or (*N*)SEEV (Wickens, 2015; Wickens et al., 2007) can compare current and nominal gaze behavior. When used to evaluate attention, the assumption is made that modeling what commonly *is* attended represents what *should* be attended. While this is reasonable for normal conditions, it may fail in error-prone or expectation-defying scenarios.

Hooey et al. (2011) evaluate SA as a ratio between actual and optimal awareness among individual situational elements, weighted by their relevance. Aspects of saliency, expectancy, and effort are not incorporated to predict likelihood of gaze, but to estimate difficulty of perception and comprehension. However, this approach often assumes a simple threshold of fixation eccentricity or duration to signify perception, and lacks quantitative calibration or validation (Fletcher & Zelinsky, 2009; Wickens et al., 2007). To understand how awareness can be inferred from gaze, large scale ground-truth labeling of SA is needed.

A variety of techniques exist to obtain such SA labeling (Nguyen et al., 2019; Stanton et al., 2013, Chapter 7). However, we believe that currently there is no suitable method for on-road, per-object awareness assessment. Physiological measures of SA lack construct specificity. One possible exception is electroencephalography (EEG), which can track attention allocation to audio (Lu et al., 2018) and detect perception of hazards, conflicts, or errors (Spüler & Niethammer, 2015; Wessel, 2012), but is not sufficiently discriminative to reliably detect single events. Self-rating and observer rating techniques are limited to aggregate measures rather than per-object assessments.

Freeze probe techniques like situation awareness global assessment technique (SAGAT) measure object-specific SA, but can only be used in simulators and measure recollection instead of awareness (de Winter et al., 2018). Recollection cannot probe unconscious/implicit awareness and suffers from inaccuracies like forgetting (Nisbett & Wilson, 1977), limiting it to partial scene probing (Gugerty, 1998). Real-time probes like situation present assessment method (SPAM) (Durso & Dattel, 2004; Strybel et al., 2016) or verbal protocol methods (Salmon et al., 2014) circumvent these issues. However, the real-time communication is intrusive and limits probe rate. Furthermore, nonchoreographed scenarios require that questions are generated real-time, as demonstrated by Sirkin et al. (2017) who automatically generated questions requiring simple yes/no and touch responses from the driver.

For this study, we build upon this idea of computer-generated queries for unchoreographed on-road driving, and extend it to (1) enable the assessment of all relevant situational elements as opposed to sampling one at a time and (2) not distract the driver visually or cognitively while driving, so that it can be applied to complex maneuvers without overloading the driver. It is applicable to any driving scenario, but we apply it to left turns on urban intersections.

To prevent dangerous distraction, the probing task was performed after crossing the intersection and parking the car. This delay meant the driver had to memorize what transpired for longer compared to freeze probe methods, which may lead to memory decay. To minimize effects of decay, we use a visual recognition task instead of a recall task. Visual detail can be encoded quite effectively. Brady et al. (2011) reviews that natural scenes can be consolidated into memory within 100–500 ms, while Lyu et al. (2020) and Choe et al. (2017) show that such encoding occurs incidentally without an attempt to memorize, which supports the idea that recognition can probe implicit as well as explicit awareness (Campodonico & Rediess, 1996). Working memory tasks have demonstrated that encoding fidelity reduces as demand increases, and that encoding multiple objects simultaneously is particularly difficult (Brady et al., 2011). Change blindness tests have demonstrated that changing vehicle presence, location, and orientation are noticed, but subtle color changes are not (Koustanaï et al., 2012). However, Konkle et al. (2010a, 2010b) also demonstrate that a recognition task allows participants to identify scenes and objects among similar decoys with high accuracy (87% and up) after briefly observing 2500 images.

For this study, we designed a simple recognition task, where the driver has to identify images of encountered road users among distractor images. Successful recognition requires that the road user was perceived explicitly or implicitly, and thus provides an indicator for Endsley’s (1995b) Level 1 situation awareness.

## Research Objective

We aimed to gain insight in drivers’ natural viewing at intersections and how well SA can be predicted from gaze metrics relative to individual road users.

The main research questions addressed were:

1. Can a recognition task be used to assess per-object awareness?
2. Can SA be predicted from gaze metrics relative to individual road users?
  - To which extent can gaze metrics predict object relevance and object recognition?
  - To which extent are foveal and peripheral vision effective in the detection of other road users?

We developed a new method measuring SA in an urban on-road driving environment, and evaluated how well a variety of object-related gaze parameters can predict recognition after left-turn maneuvers. Driver gaze was related to object location, type, and relevance for safety, using the road scene perception of our experimental vehicle. We then assessed the distribution of central and peripheral detections of safety-relevant and -irrelevant road users.

## Method

We designed an experiment in which participants drove a Toyota Prius instrumented for automated driving and road scene perception (Ferranti et al., 2019) and eye tracking. The drivers (manually) performed left turns at multiple crossroads while the vehicle collected gaze behavior in relation to other road users. After each turn the driver parked the vehicle, and an object recognition task was performed to measure awareness of other road users encountered on or near the intersection. In subsequent sections, we detail the tracking of gaze and road users, the implementation of the recognition task, and the experimental procedures.

### Tracking of Gaze and Other Road Users

**Figure 1:**
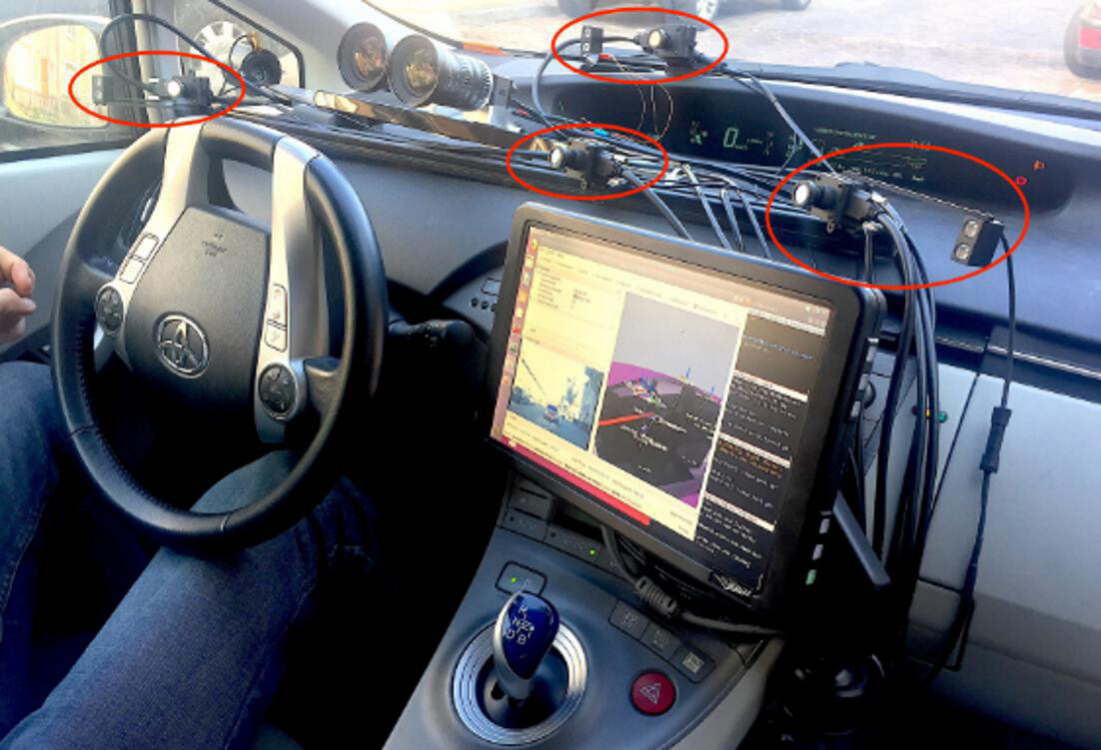
Interior of the vehicle showing the four eye-tracker cameras encircled in red, and the built-in display used for the post-drive recognition task.

We used a four-camera Smart Eye Pro dx 5.0 eye tracker (software version 8.2) running at 60 Hz with a gaze accuracy down to 0.5°. The vehicle interior and setup are shown in Figure 1.

**Figure 2:**
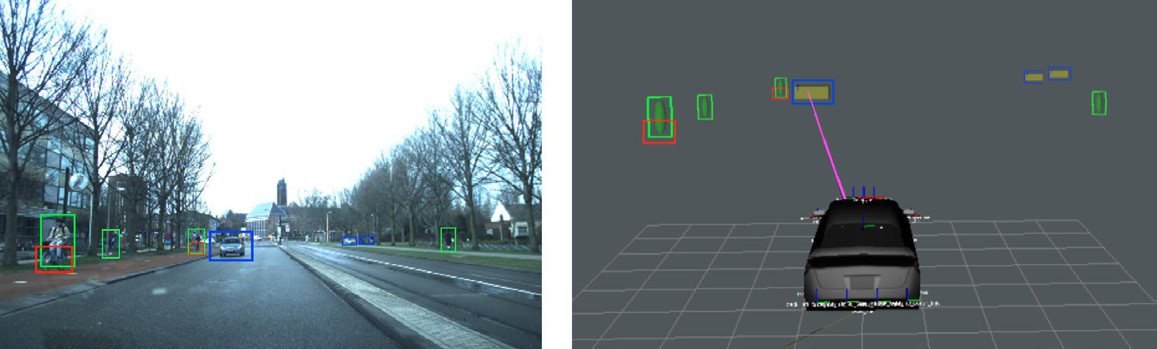
Left: road scene with highlighted detections. Right: 3D visualization of the detected objects. The magenta ray visualizes the driver’s gaze.

The road was observed at 10 Hz using two forward-facing IDS 2.3-megapixel cameras mounted near the top-center of the windshield and placed 22 cm apart for obtaining a dense stereo depth image over a visual angle of 62°. Detection of other road users was performed using a single-shot detector (Liu et al., 2016). Using the depth-image, detections were projected in 3D using the 15 percentile distance of all pixels inside the detection bounding box. Gaze analysis was limited to the horizontal component to reduce tracking artifacts caused by vehicle pitch motion at the cost of losing some specificity in the association of gaze angles to road users. After correcting for vehicle ego-motion, the road users were tracked in 3D space using a Kalman filter, and up-sampled to 60 Hz using linear interpolation in synchronization with the eye tracker. For each tracked object, the image with the largest bounding box was stored for use in the recognition task. Each image was made 20% larger than the bounding box to include some of the surrounding environment. It was then scaled to 200 × 200 pixels and normalized in brightness and contrast to reduce optical differences between real and dummy images. Information was integrated as visualized in Figure 2.

### Recognition Task

**Figure 3:**
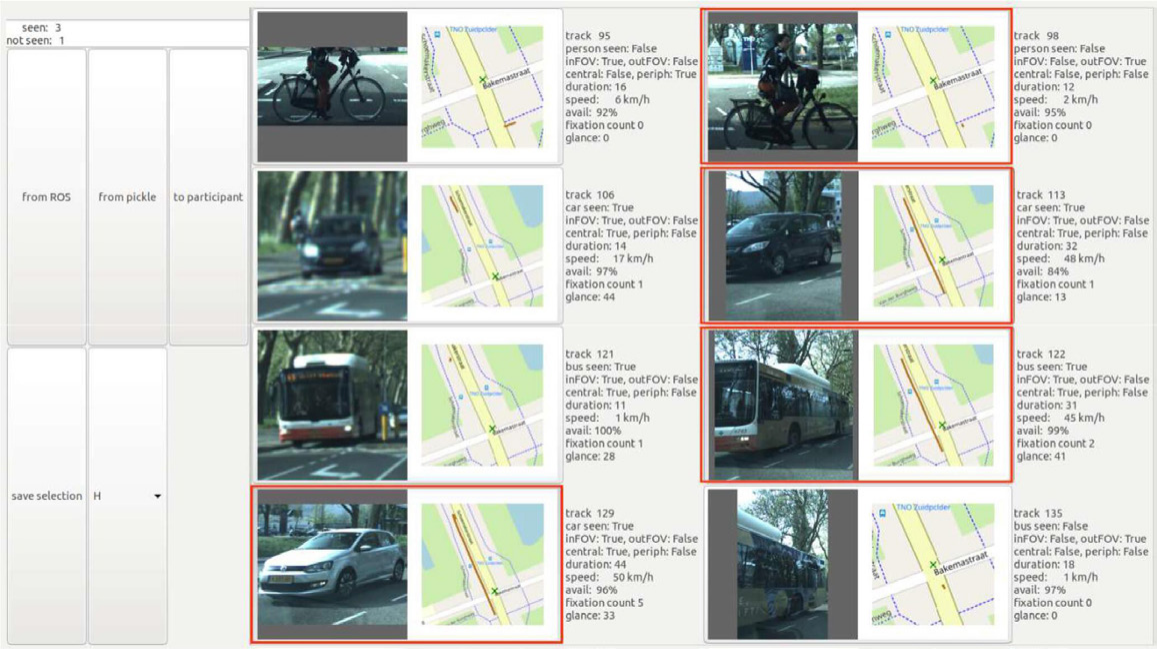
Example image of the preselection GUI in which the experimenter selected suitable object images to be presented to the participant. The GUI allowed scrolling up and down for additional images. GUI = graphical user interface.

After each intersection, the driver parked the vehicle, and the recognition task was performed on the vehicle center display. The experimenter first prepared a selection among the road-user images, which the vehicle had collected during the maneuver. The procedure was to avoid parked vehicles and blurry or partial images, and to select the clearest image/trajectory whenever multiple images were available for the same road user. The preselection graphical user interface (GUI) is shown in Figure 3. Each road user was represented with a color image, its traveled path over the intersection, and a summary of the road user properties (e.g., type, speed) and gaze parameters.

**Figure 4:**
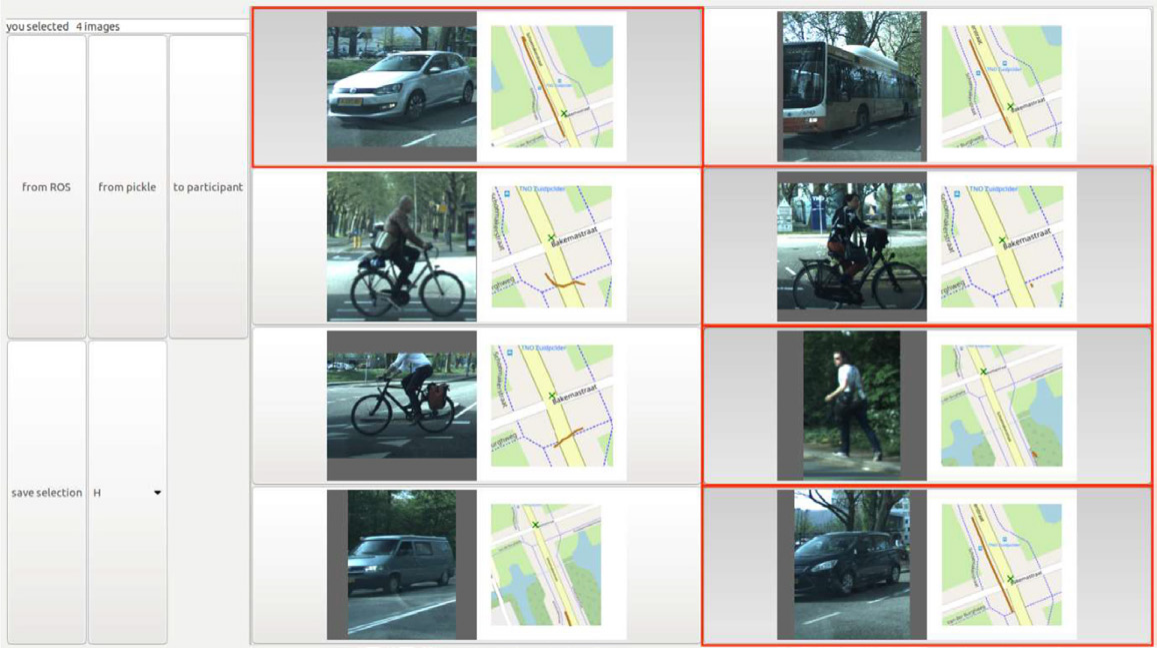
Example image of the GUI in which the participants selected images of the objects they recognized. Four images are selected in this example. The GUI allowed scrolling up and down when more than eight images were presented. GUI = graphical user interface.

The selected images were then presented to the participant. Dummy images from an earlier session at that same intersection were added to discourage guessing. Collectively, these dummy images consisted of 53 cars, 17 bicycles, six pedestrians, one bus, four trucks, one motorcycle, two construction vehicles, and one dog. The participants were made aware that the task contained both road users they just encountered and dummy objects, and each dummy image was used once for each participant. The gaze details and vehicle data were not shown to the participant. The participant GUI is shown in Figure 4.

### Procedures

The criteria to participate in this study were being a staff member or student of the department (for insurance reasons), having a driving license, and having driven an automatic transmission at least once before. Fourteen male drivers participated, of which one was excluded from the analysis because the drive was not recorded correctly. The remaining 13 were aged between 24 and 57 years (*M* = 28.8, *SD* = 8.8). One had a license for 1–5 years, nine for 5–10 years, and three for more than 10 years. Five participants drove less than once a month, four drove once a week, three drove 1–3 times a week, and one participant drove every day. Four participants did not wear any visual aids, four wore glasses, and four wore contact lenses. The research was approved by the Ethics Committee of the TU Delft. All participants read and signed an informed consent form prior to the experiment. They received a box of chocolates for their participation.

All participants were informed on the purpose of this study prior to participation and had a technical understanding of the used technology, but not of the recognition task or its implementation. Eye tracker calibration typically resulted in 1.2° accuracy and was repeated when accuracy exceeded 2° for at least one eye. They were navigated by the experimenter. Upon approaching each intersection, data recordings were started. The participants were asked to make a left turn and then safely stop or park the car at the first opportunity. They were asked (and asserted by the experimenter) to not look at the display while the recognition task was prepared. Once ready, the participants performed the recognition task without time constraint. The participants then returned to the main road and the procedure was repeated for all intersections. The participants returned to the starting location, where they completed a personal information questionnaire, rated the difficulty of the recognition task, and indicated if they used the images, maps, or both for their decisions.

**Figure 5:**
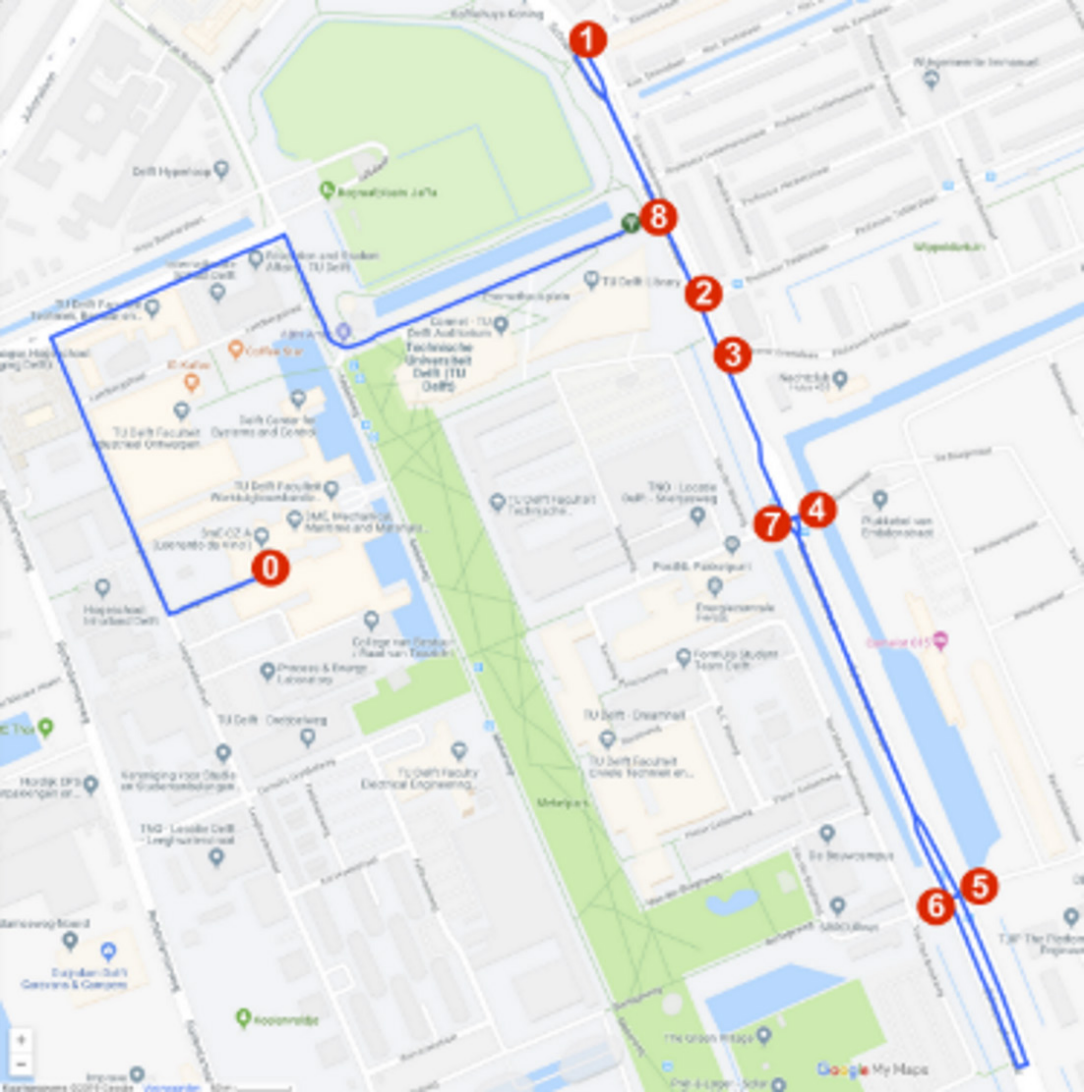
Map of the experiment driving route and the locations and order of the maneuvers.

**Figure 6:**
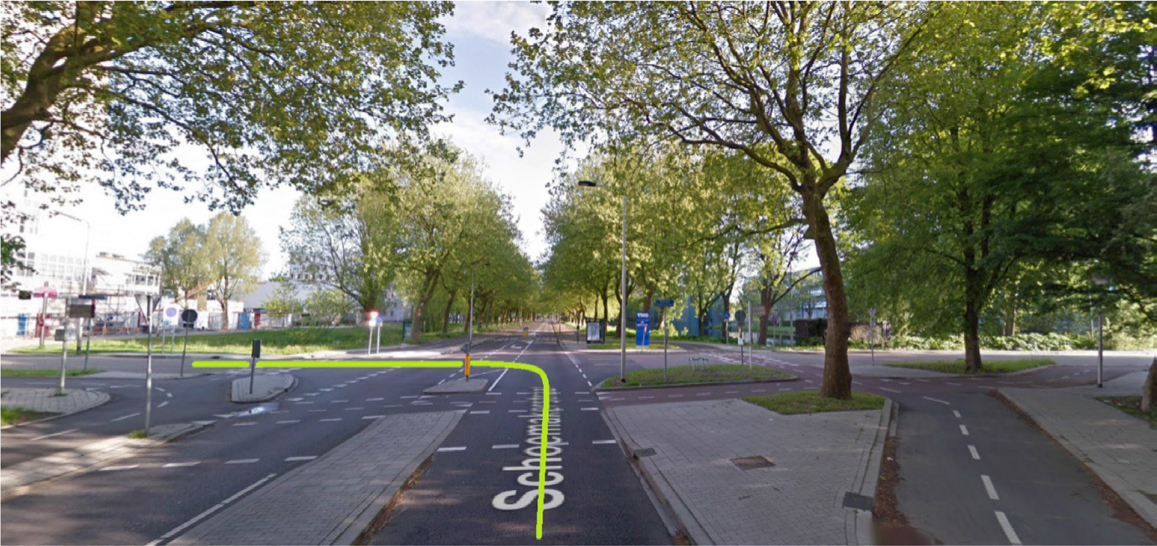
One of the intersections. The green line corresponds to maneuver 6. (Google Street-View, 2019).

The driven route is shown in Figure 5. Five intersections on the Schoemakerstraat in Delft were selected for their complexity, similarity, and presence of traffic throughout the day. Three intersections were T-junctions that were passed once, and two were crossroads that were passed twice from opposite directions. The drivers had to give priority to oncoming traffic on the main road, to cyclists on the two-way bicycle path, and to pedestrians on the sidewalk. A typical intersection is shown in Figure 6. Maneuver 1 was an additional right turn used to practice the recognition task and was not analyzed.

### Filtering and Merging Objects

**Figure 7:**
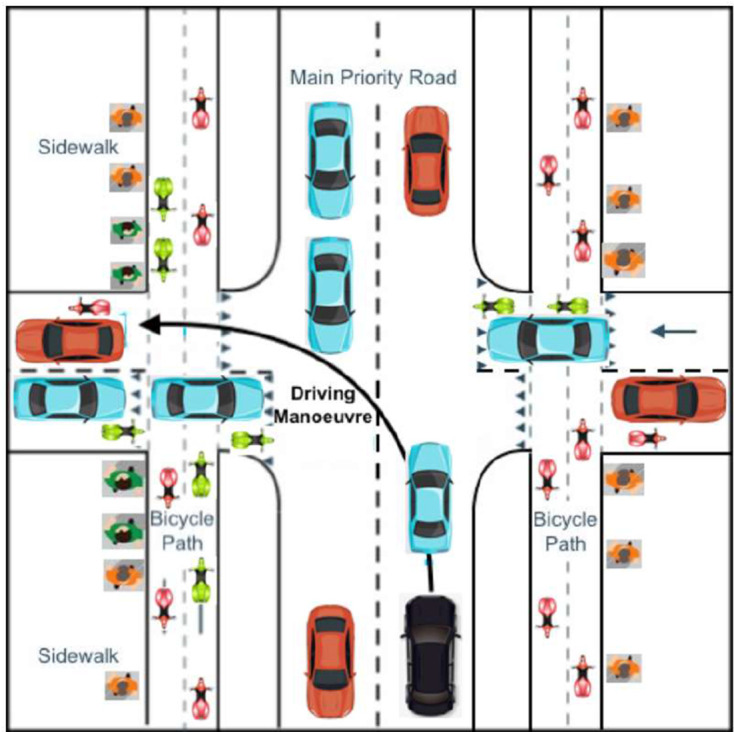
Schematic visualization of how objects were annotated according to their position and movement direction: relevant road users (blue/green) and irrelevant road users (red) and the car driven by the participant (black).

After the experiment, the collected data were filtered manually. Split or duplicate tracks of the same road user were merged. All road users were annotated as being relevant or irrelevant to the driving maneuver. The second author subjectively judged if a driver would want to monitor each road user for the purpose of driving at any time during the maneuver. A road user was considered relevant if the annotator felt that the participant had to give priority or should obtain priority at the intersection. Road users that left the intersection before the participant arrived at the intersection or were still well away when the participant left the intersection were regarded as irrelevant. Road users on the sidewalk or the bicycle lane on the right side of the intersection were annotated as irrelevant. Road users trying to enter or cross the main priority road before the participant passed were annotated as relevant. An overview of the possible road users encountered and how they were annotated is shown in Figure 7.

### Gaze Metrics

The following parameters were analyzed.

#### Gaze eccentricity

The angle between the direction of the driver’s gaze and the vector from the gaze origin to the center of a road user (width of the road user is ignored). Only the horizontal component is used in this study.

#### Minimum gaze angle

The smallest gaze eccentricity toward a road user throughout the period this road user was tracked by the vehicle. Saccades (angular rates beyond 35°/s) are ignored.

#### Total glance duration within visual field regions

The summed duration of all fixations occurring while the gaze eccentricity falls within one of the following regions (Duchowski, 2007): foveal view (<2°) where highest visual acuity is obtained, near-foveal view (2–5°) in which objects are commonly recognizable, central view (5–10°) up to which acuity and color sensitivity degrade linearly, near-peripheral view (10–30°) and far-peripheral view (>30°).

Huestegge and Böckler (2016) compared saccade behavior during the detection of critical and moderate hazards in static scenes and found that more critical hazards are detected earlier, at larger peripheral angles, and with shorter fixation durations preceding the first saccade to these hazards. We, therefore, evaluated the related metrics.

#### First saccade angle

The visual angle between start and end of the first saccade that lands within 2° of the object.

#### First saccade time

The time over which the object has been tracked by the vehicle before a first saccade lands within 2° of the object. Any saccade landing on the object before it was detected by the vehicle is not observable and thus ignored.

#### Duration preceding fixation

The duration of the fixation that preceded the first saccade landing within 2° of the object.

We also observed the following parameters that are often considered to assess situation awareness. They were excluded from regression analysis because they are structurally correlated to the total glance duration within 2°. Instead, simple effects are reported.

#### Number of fixations

The number of fixations occurring while the object is within 2° of the gaze vector. It is equivalent to the number of saccades.

#### Mean glance duration

Total glance duration within 2° divided by the number of fixations.

### Binary Logistic Regression

A binary logistic regression was performed to test if the gaze parameters can predict the participants’ selections in the recognition task. We also tested if gaze parameters can predict object relevance. To account for subject dependencies, both models use participant as a random variable for the intercept.

We had to address missing values for saccade-related variables, which are defined only when they land within 2° of an object. List-wise elimination is not desired since we want a prediction even for objects that were not glanced upon directly. Instead, we adopted Cohens’ dummy-variable adjustment (Cohen & Cohen, 1983). This approach is not generally recommended, as it may induce bias from conditional inclusion (Allison, 2009). In our case, however, such a bias is not a concern as the missing values are a structural property of the model. The model structure thus becomes:

$$Y=b_{0\;j}+b_{1}X_{1}+Z\left(b_{2}X_{2}\right)+e$$

Here, *b*_0_ *_j_* represents the intercept, which is allowed to vary among participants; *X*_1_ are total glance durations for the five eccentricity ranges; *X*_2_ are saccade-related variables; and *Z* is the dummy variable where *Z =* 1 when saccades are available and 0 otherwise.

## Results

In the recognition task, participants had to select images of road users they just encountered. A total of 91 intersection crossings were collected and 1824 images were presented to the 13 participants in total. On average, there were 8.2 images of real objects and 11.8 dummy images per intersection per participant. The number of images presented to the driver varied with a standard deviation of 6 and ranged between 5 and 34. It took 30 s on average to park the car after leaving the intersection, and another 30 s for the experimenter to prepare the recognition task. Participants took approximately 80 s to select images. The questionnaire showed that the participants rated the difficulty of the recognition task as 8.6 on a scale of 1 to 10, with 1 being really easy and 10 being really difficult. Due to dropped messages in the recordings, 7% of the gaze data could not be re-associated to the recognition task images and were omitted from the gaze-related analysis.

### Selection of Images

Table 1 shows how often participants selected images of real and dummy objects and provides an indication of response bias and sensitivity. The odds of selecting an image was 5.7 times higher for real compared to dummy images (95% CI = 4.3, 7.6). Only 29.1% of real images were selected. Since no unsafe driver behavior was noted, the remaining 70.9% does not necessarily represent overlooked road users. Hence, recognition rates reported in this study must underestimate the actual SA and our recognition task can thus not fully address research question 2.

**Table 1:** Contingency Table of the Selected Images of Real Objects and Dummy Objects, as Well as Relevant and Irrelevant Objects

	Selected	Not Selected
Real images	218 (29.1%)	532 (70.9%)
Relevant objects	144 (36.1%)	255 (63.9%)
Irrelevant objects	74 (19.4%)	307 (80.6%)
Dummy images	72 (6.7%)	1002 (93.3%)

**Figure 8:**
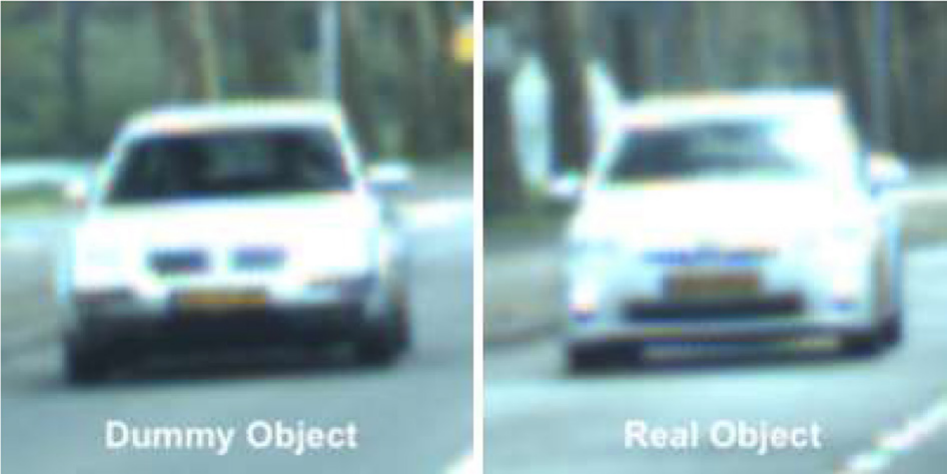
Left: an example of a selected dummy image—a silver Volkswagen. Right: a not selected real image actually encountered—a silver Toyota.

The 93.3% not selected dummy objects suggest that the participants adopted a select-only-when-certain philosophy. While the 72 selected dummy images could result from guessing, some may have been confused with real objects: 13.7% of these dummy images shared a close resemblance to a real image, similar to Figure 8; and 24.7% had an approximate resemblance to a real image of same type, approximately sharing color and/or shape. Jointly this suggests that selected images indeed represent perceived road users.

Relevant (real) road users were recognized more often (36.1%) than irrelevant ones (19.4%). This interaction effect is significant (χ^2^(1) = 26.06, *p* < .001, φ_c _= .186) with an odds ratio of 2.3 (95% CI = 1.7, 3.3).

### Minimum Gaze Angle

Table 2 shows the number of real road users divided into the different object classes and minimum gaze angles; 27.2% of the relevant and 72.2% of the irrelevant road users were never fixated upon within 2°. Similarly, 40.1% of the recognized and 53.7% of the not recognized road users were never fixated upon within 2°. These values are surprisingly large and indicate that a considerable number of objects were perceived without ever receiving a direct fixation. Cars had the lowest recognition rate (26.2%) despite being the most common. Buses were recognized the most (61.5%), followed by motorcycles (44.4%) and pedestrians (39.0%). When only considering relevant road users, pedestrians were recognized the most (64.3%). Minimum gaze angle interacted significantly with recognition (χ^2^(4) = 16.07, *p* = .003, φ_c _= .151), suggesting that higher eccentricity leads to poorer recognition. Minimum gaze angle also interacted with relevance (χ^2^(4) = 151.35, *p* < .001, φ_c _= .463), suggesting that relevant objects are monitored more closely compared to irrelevant ones. Road user type also interacted significantly with recognition (Fisher’s exact test = 15.13, *p* = .020, φ_c _= .151) and relevance (Fisher’s exact test = 51.58, *p* < .001, φ_c _= .266).

**Table 2:** Number of Real Road Users Observed at Various Minimum Gaze Angles, for All Objects (Left) and Those Selected During the Recognition Task (Right). The “Other” Category Comprises One Dog and Two Excavators

	All Objects (Minimum Gaze Angle)						Recognized Objects (Minimum Gaze Angle)					
	*N*	<2°	2–5°	5–10°	10–30°	>30°	*N*	<2°	2–5°	5–10°	10–30°	>30°
Car	409	257	62	39	40	11	107	76	14	9	6	2
Relevant	241	191	23	15	9	3	81	63	9	6	2	1
Irrelevant	168	66	39	24	31	8	26	13	5	3	4	1
Bicycle	184	67	24	27	57	9	54	31	8	5	8	2
Relevant	83	48	11	9	13	2	39	29	5	3	2	0
Irrelevant	101	19	13	18	44	7	15	2	3	2	6	2
Pedestrian	77	17	12	10	32	6	30	10	7	3	9	1
Relevant	14	7	2	2	3	0	9	5	2	2	0	0
Irrelevant	63	10	10	8	29	6	21	5	5	1	9	1
Bus	13	7	2	2	2	0	8	4	2	2	0	0
Relevant	5	5	0	0	0	0	3	3	0	0	0	0
Irrelevant	8	2	2	2	2	0	5	1	2	2	0	0
Truck	11	3	2	1	4	1	3	1	0	0	2	0
Relevant	6	3	1	0	2	0	2	1	0	0	1	0
Irrelevant	5	0	1	1	2	1	1	0	0	0	1	0
Motor	9	4	1	3	1	0	4	2	1	1	0	0
Relevant	4	3	0	1	0	0	2	2	0	0	0	0
Irrelevant	5	1	1	2	1	0	2	0	1	1	0	0
Other	3	0	0	1	2	0	1	0	0	1	0	0
Relevant	0	0	0	0	0	0	0	0	0	0	0	0
Irrelevant	3	0	0	1	2	0	1	0	0	1	0	0
Total	706	355	103	83	138	27	207	124	32	21	25	5
Relevant	353	257	37	27	27	5	136	103	16	11	5	1
Irrelevant	353	98	66	56	111	22	71	21	16	10	20	4

**Figure 9:**
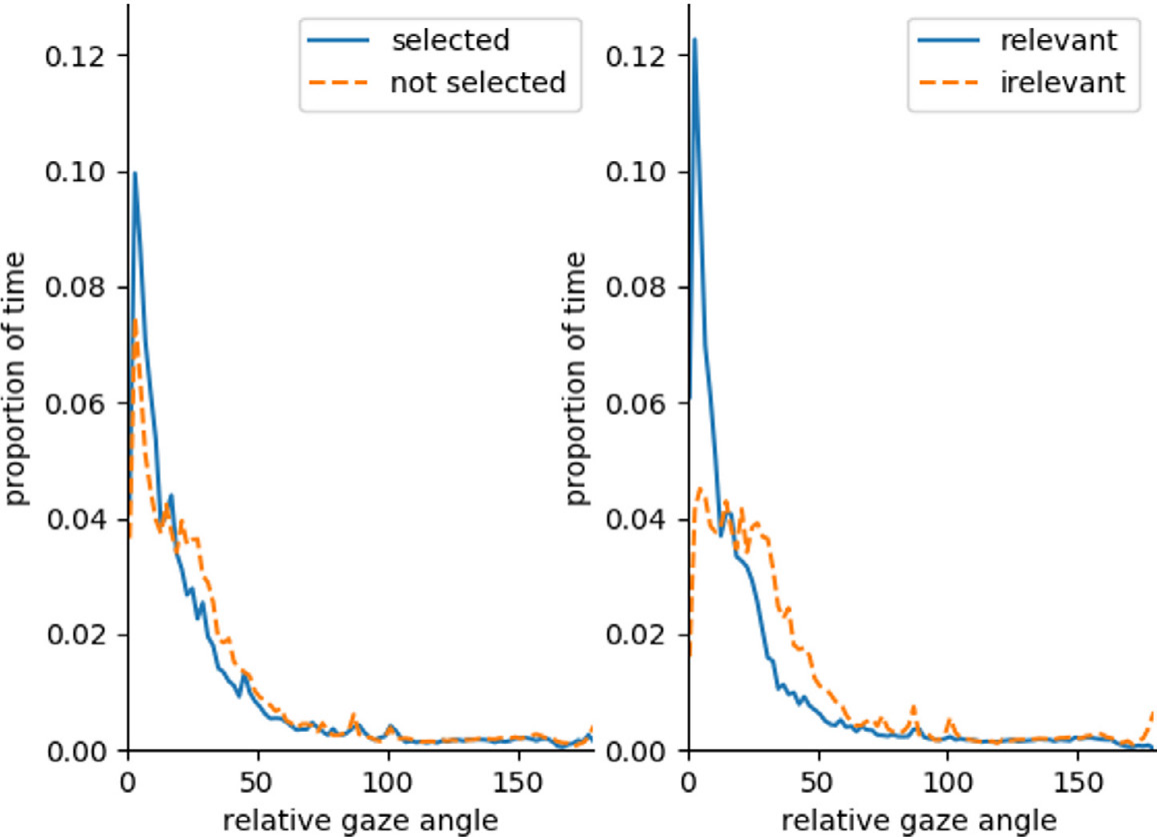
Distribution of relative gaze angle for (not) selected (left) and (ir)relevant (right) road users over the period they were detected by the vehicle cameras.

Figure 9 shows the distribution of gaze angles over the duration that an object was tracked by the car, averaged over all tracked objects. Driver gaze dwelled closer to relevant compared to irrelevant road users. A similar effect is not as clear between selected and not selected road users.

### Fixation Parameters

**Figure 10:**
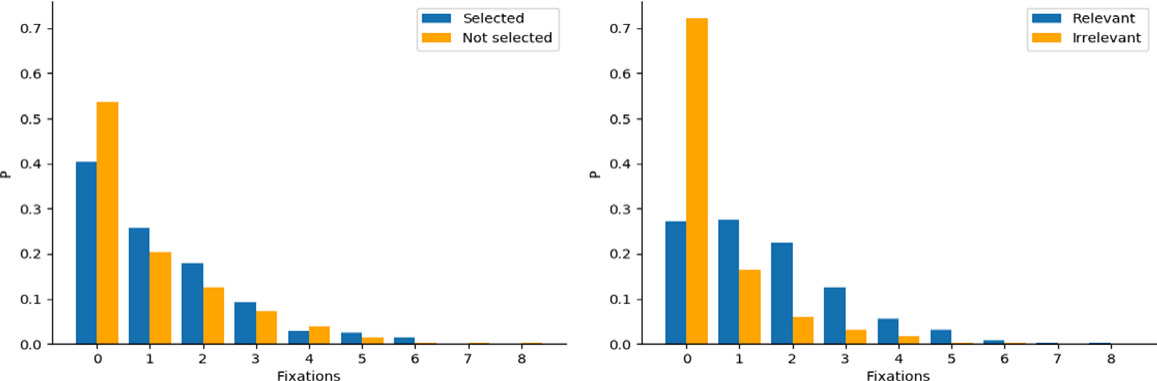
Distribution of fixations (<2°) per road user, comparing selection in the recognition task (left) and relevance (right).

**Figure 11:**
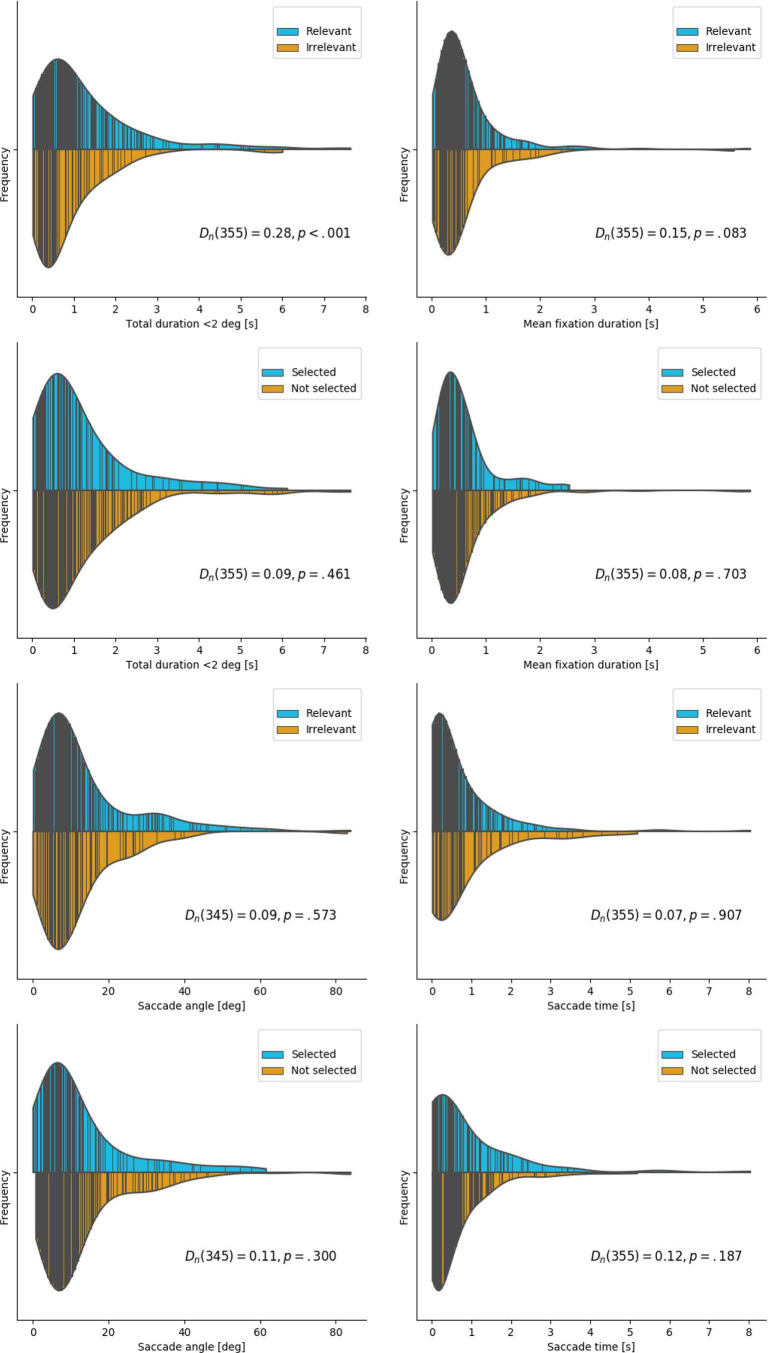
Distributions and Kolmogorov–Smirnov tests for a selection of gaze parameters, comparing relevance and selection in the recognition task.

Table 3 compares fixation parameters for relevant versus irrelevant and selected versus not selected road users. Figures 10 and 11 illustrate the distribution shapes for a selection of gaze parameters comparing relevance and selection in the recognition task. All are right-tailed. The Kolmogorov−Smirnov tests in Figure 11 represent a nonparametric statistic of similarity between the paired distributions. Only total glance duration with fixation angle <2° yielded a significantly different distribution between relevant and irrelevant road users. To avoid dependencies among participants and reduce non-normality of the distribution of the residuals, we used participant-averaged paired *t*-tests. Relevant road users received 1.14 more fixations compared to irrelevant road users. Selected road users received 0.41 more fixations compared to not selected road users. Mean fixation duration did not differ significantly in either case.

**Table 3:** Fixation Parameter Mean (μ) and Standard Deviation (σ) as Function of Relevance (Top) and Being Selected (Bottom)

	Relevant		Irrelevant		*T*(12)	*p*
	μ	σ	μ	σ		
Number of fixations <2°	1.59	0.51	0.45	0.26	9.653	<.001
Total fixation duration <2° (ms)	955	309	237	127	9.318	<.001
Mean fixation duration (ms)	658	264	685	676	−0.134	.895

	Selected		Not Selected		*T*(12)	*p*
	μ	σ	μ	σ		
Number of fixations < 2°	1.34	0.51	0.93	0.33	2.341	.037
Total fixation duration <2° (ms)	833	519	536	197	1.944	.076
Mean fixation duration (ms)	633	272	640	257	−0.113	.912

### Binary Logistic Regression

Table 4 shows the classification performance for the recognition and the relevance models. Model accuracy was compared to intercept models, which obtained an accuracy of 70.7% by simply predicting that no objects were selected, and an accuracy of 50.0% by predicting that all objects were relevant. Both models differed significantly from their intercept models (Recognition: χ^2^(1) = 37.64, *p* < .001, φ_c _= .231. Relevance: χ^2^(1) = 152.49, *p* < .001, φ_c _= .465), where accuracy increased by only 2.12% to 72.80% for the recognition model and by a more substantial 23.09% to 73.09% for the relevance model.

**Table 4:** Classification Performance of the Logistic Regression Models

Intercept Models				Parameterized Models			
Relevance		Recognition		Relevance		Recognition	
Predicted	True	False	True	False	True	False	True	False
Observed								
True	353	0	0	207	238	115	44	163
False	353	0	0	499	75	278	29	470

Table 5 provides the exponentials and significance of the model parameters. The odds for a road user being relevant increases significantly with gaze duration in relative gaze angle ranges of <2°, between 2 and 5° and between 5 and 10°. Gazes at larger angles do not seem to discriminate between relevant or irrelevant road users. The odds for a relevant road user also increases slightly when the first saccade within <2° has a larger angle. Timing of the first saccade or the duration of its preceding fixation do not help to discriminate relevance of road users.

**Table 5:** Parameters of the Logistic Regression Models

	Relevant					Recognized				
	Exp(b)	*t*	*p*	5% CI	95% CI	Exp(b)	*t*	*p*	5% CI	95% CI
Intercept	.390	−3.629	<.001	.234	.649	.262	−4.274	<.001	.141	.484
Duration <2° (s)	5.452	3.024	.003	1.813	16.398	1.424	1.591	.112	.921	2.204
Duration 2–5° (s)	2.658	3.273	.001	1.479	4.778	.956	−.157	.875	.544	1.679
Duration 5–10° (s)	2.541	4.188	<.001	1.641	3.934	1.995	3.153	.002	1.298	3.067
Duration 10–30° (s)	1.094	.741	.459	.862	1.390	.946	−.402	.688	.720	1.242
Duration > 30° (s)	.693	−1.574	.116	.439	1.095	.929	−.444	.657	.670	1.288
1st Saccade angle (°)	1.049	2.756	.006	1.014	1.085	1.001	.132	.895	.982	1.021
1st Saccade time (s)	.901	−.613	.540	.646	1.257	1.243	1.334	.183	.903	1.711
Preceding fixation (s)	1.087	.345	.730	.677	1.744	1.361	1.217	.224	.828	2.239

The odds of recognizing a road user increases significantly only when the relative gaze angle spends more time between 5° and 10° from the road user, and a similar effect for gaze <2° does not reach significance (*p* = .11). Since this model used nine parameters to only achieve a 2% accuracy improvement over the intercept model, the relevance of these results is limited.

## Discussion

This study set out with two objectives: to evaluate if the developed recognition task can provide useful labeling of per-object situation awareness and to evaluate whether awareness of other road users can be predicted from gaze behavior in relation to these objects.

### Suitability of the Recognition Task

Relevant road users were recognized more often than irrelevant road users, which is in line with Moore and Gugerty (2010). However, drivers recognized only 29.1% of all road users, 36.1% of the relevant road users, and 40.0% of relevant road users that were fixated <2°. These unexpected low recognition rates mean that the current implementation of the recognition task is only partially successful in labeling situation awareness. Below, we analyze the limited recognition rate and provide suggestions to adapt the task to enhance recognition.

While the vehicle processed all video and gaze measurements in real time, 60 s elapsed between finishing the maneuver and performing the recognition task. This time was needed for the participant to stop the vehicle and for the experimenters to select images for the recognition task. This delay may have contributed to the low recognition rate. Humans are normally poor in remembering details of past events with a rapid decay of information in working memory, which is limited to around 30 s (Nisbett & Wilson, 1977). In contrast, Endsley suggests that SAGAT-like techniques do not suffer much from memory decay up to 3 min, provided that the participant is experienced (Endsley, 2019, 1995a). Delays below 30 s should be feasible if the selection of suitable images is automated, and a location is reserved after each intersection for faster parking.

Second, it is possible that our image representation differed too much from how situations are encoded by experienced drivers. Performing a left turn on a busy priority road is relatively demanding. Working memory makes trade-offs between the quantity of stored items and their fidelity. The more road users we encounter, the fewer details about them we can store, and task-irrelevant features are the first to be dropped (Brady et al., 2011). Our images contained little task-relevant context. Although the maps provided some spatial context, all participants reported to primarily base their decisions on the images. A possible improvement would be to show more environment in the images, and project the traveled paths into the images instead of the separate map.

### Suitability of Gaze Behavior

We parameterized gaze behavior relative to nearby road users. Such gaze parameters may be useful in driver attention and awareness modeling and driver support system design.

Gaze behavior could predict object relevance with an accuracy of 73%, where relevant objects were more often fixated <2°, with larger first saccade angles, and with a higher gaze duration up to 10° eccentricity. This illustrates that relevant road users are kept more within the useful field of view compared to irrelevant road users. Mean first saccade amplitude was 12.6°, with a strongly skewed distribution well into the 30° region. This suggests that peripheral vision was effectively used to direct gaze to relevant road users.

While the first saccade angle contributed significantly to the relevance model, timing of the first saccade and duration of the preceding fixation did not. This difference with the findings of Huestegge and Böckler (2016) could mean that the usefulness of saccade parameters is limited to hazards. Saccade-related parameters may also become more useful when studied in relation to events like changes in the road users’ behavior instead of their first appearance in the driving scene.

Gaze behavior was not very effective in predicting outcomes of the recognition task. One explanation is that the forgetting aspect could not be captured by our model. We expect that improved methods and simpler conditions can enhance recognition rate and further clarify the relation between recognition and gaze. Meanwhile, the recognition task did provide useful insights; 18% of the road users that never entered the useful field of view (<10°) were still selected in the recognition task, highlighting the importance of peripheral vision (Wolfe et al., 2017). Hence, we strongly recommend that perception models incorporate more than fixation location in their parameterization. Our findings may also provide guidance for designing a system alerting drivers toward other road users, which may be unseen. From Table 2, we estimate how frequently such a support system might alert drivers. Our data set includes 8.2 road users per intersection of which 4.4 are relevant to the maneuver. When a gaze-aware system alerts to every peripheral (>10°) road user, the driver receives 1.8 alerts per intersection. Since drivers recognized 18% of the peripherally observed road users, they would have been aware of at least 0.33 alerts beforehand. When only responding to relevant peripheral road users, the driver receives only 0.35 alerts per intersection and would be aware of only 0.07 alerts beforehand.

## Limitations and Future Recommendations

The recognition task can be improved to obtain complete rather than partial labeling of per-object situation awareness in complex unstructured maneuvers. The main limitations—delay before start of the test and task visualization—are likely to be overcome. Better object detection and especially more robust tracking could circumvent the need for manual preselection of candidate images, and thus reduce the delay between actual encounters and the recognition task. Further improvements may be achieved by reducing the number of test images as recommended by Gugerty (1998) or by associating gazes more selectively to a single road user, for instance, through including the vertical gaze component or with a Dynamic Markov random field model (Jiang et al., 2018) or a Bayesian likelihood model (Schwehr & Willert, 2017).

After such improvements, the potential of the recognition task as a variant of freeze probe methods can be explored. Benefits may emerge in simpler maneuvers or in the better controlled simulator environment. Improvements of the recognition task’s visualization may better suit the driver’s encoding of situational information. To improve retrieval for recognition, road users could be depicted in the road scene (Hollingworth, 2006), and road-user motion could be encoded using video, animation or multiple images.

Finally, to more closely examine time-critical attention allocation, it may be interesting to study gaze behavior relative to road user actions (such as a change in traveled path or speed) in addition to the aggregate road user parameters used in this study.

## Key Points

- Gaze relative to surrounding traffic was compared to a recognition task during on-road left-turn maneuvers.
- Gaze behavior could predict object relevance where relevant road users were kept longer in the useful field of view.
- Drivers recognized 18% of the peripherally observed road users, which suggests that perception models should consider more than foveated vision.
- Driver feedback can become more selective when driver awareness of individual road users is monitored.
